# Supplementary material for: Chromothripsis during telomere crisis is independent of NHEJ, and consistent with a replicative origin
Source: Genome Res. 2019 May;29(5):737–49. doi: 10.1101/gr.240705.118 (PMC6499312; doi:10.1101/gr.240705.118)
Supplement: Supplemental Material [file supp_gr.240705.118_Supplemental_file_1.zip › contigs/annotated_contigs/DB114/contig.2.DB114_length_621_mean_cov_9.4847020934.docx]

**DB114_length_621_mean_cov_9.4847020934**

```
CACAGTATCTTATCTTCACTTTTAACTCTGTAAAGTCCAAAATCAGGCATAATCTCAGTAATTTTTATCCACTAAACTTCCTTCTGTAG
 >chr10:85172658-85173027 + E=2e-207
ATGTTTCTATCCTTGGAAATATTTATGGGTACTTTAATGTGAATTAGGAAGAGAGCTTTCCTGACATTTTCCCACAAATTATCAGCCTG

CTATGCAAGCAAGTAGAAAGTTACACAATGAAAATGAAGATGCTAGGTTTCCAAGCCTAAGGATCAAATTTAGAAATATGAGTATATTA

TTTACCAACTAAAATTGAGAAAATGTTACATAGAAATTCTAAGATCAATAAGCAGATTGATCTGCATGTAAAAGATGAATATAAGCAAT

AACAATCATAG|AG|CCTCCTGAGTAATTAGGATTACAGGCAGGCATCACCACGCCCAGCTAATTTTTGTATTTTTAGTAGAGACGGGG
            >chr12:7463408-7463662 - E=4e-138
TTTCACTACGTTGGCCAGGCTGGTCTCAAACTCCTGACCTCAAGTGATCCACCCACCTCAGCCTCCCAAAGTGCTGGGATTACAGGTGT

GAGCCACCGCACCCAGCTATTAGGTAATTCTTTTCTGAGTAAGAGCCCAGATCAACTGACAGTGTTCCTGAGTCCTTGGAGGCAAAGAG
```
